# The challenge of making the right choice: patient avatars in the era of cancer immunotherapies

**DOI:** 10.3389/fimmu.2023.1237565

**Published:** 2023-08-10

**Authors:** Charlotte Kayser, Annika Brauer, Sebens Susanne, Anna Maxi Wandmacher

**Affiliations:** ^1^ Group of Inflammatory Carcinogenesis, Institute for Experimental Cancer Research, University Hospital Schleswig-Holstein (UKSH), Kiel University, Kiel, Germany; ^2^ Department of Internal Medicine II, University Hospital Center Schleswig-Holstein, Kiel, Germany

**Keywords:** organoids, organotypic tissue slice culture, organ-on-a-chip, patient-derived xenografts, tumor (immune) microenvironment, precision oncology, translational oncology

## Abstract

Immunotherapies are a key therapeutic strategy to fight cancer. Diverse approaches are used to activate tumor-directed immunity and to overcome tumor immune escape. The dynamic interplay between tumor cells and their tumor(immune)microenvironment (T(I)ME) poses a major challenge to create appropriate model systems. However, those model systems are needed to gain novel insights into tumor (immune) biology and a prerequisite to accurately develop and test immunotherapeutic approaches which can be successfully translated into clinical application. Several model systems have been established and advanced into so-called patient avatars to mimic the patient´s tumor biology. All models have their advantages but also disadvantages underscoring the necessity to pay attention in defining the rationale and requirements for which the patient avatar will be used. Here, we briefly outline the current state of tumor model systems used for tumor (immune)biological analysis as well as evaluation of immunotherapeutic agents. Finally, we provide a recommendation for further development to make patient avatars a complementary tool for testing and predicting immunotherapeutic strategies for personalization of tumor therapies.

## Introduction

Immunotherapy has emerged as an important pillar in cancer therapy comprising multiple strategies, e.g. cell-based approaches as chimeric antigen receptor T cells (CAR T cells) (1–4) or tumor infiltrating lymphocytes (TIL) (5), immune checkpoint inhibitors (ICI) (6–13), oncolytic viruses (14) and tumor vaccines (15). However, despite promising preclinical data, only a very low percentage of oncological treatments reach phase III trials or even clinical application (16, 17) and even those strategies that have entered clinical routine often exert less pronounced anti-tumor effects than observed in model systems. In addition, clinicians are faced with great heterogeneity in terms of patient responses to therapy even if levels of predictive biomarkers (e.g. specific mutations or immunohistochemical staining of protein biomarkers) are comparable. This highlights the limitation of personalizing treatment strategies solely based on genomics and single biomarkers as well as the need for valid co-clinical testing systems. Functional drug testing in those co-clinical models representing the individual tumor biology of a patient as accurately as possible (also termed “patient avatars”) to predict the individual susceptibility to drugs appears as a desirable approach to truly personalize patient treatment (18). Increasing efforts are therefore made to improve preclinical tumor models in order to optimally represent the complex and dynamic interplay between tumor cells and the immune system, especially in the tumor microenvironment (TME) of solid and hematologic malignancies. Irrespective of whether the model system is used for tumor immunological studies or individualized therapy prediction, an optimal patient avatar needs to reflect intra- and intertumoral heterogeneity (19) and comprise the entire tumor (immune) microenvironment (T(I)ME) (20–22). Particularly, to test immunotherapeutic strategies, the whole spectrum of innate and adaptive immune cells should be present in the patient avatar to mimic the direct and indirect cellular interactions of tumor cells and all stromal (cell) components of the tumor.

## Tumor model systems and patient avatars

### 2D tumor cell models

Two-dimensional (2D) tumor cell models comprise established and often immortalized cell lines or primary cell cultures directly established from fresh tumor material. Established cancer cell lines derived from solid tumors, leukemias and lymphomas have been extensively used for basic cell biology experiments and drug discovery since the early 1950s (23). As these cells grow in monolayers, culture maintenance is comparatively simple, inexpensive and analyses (including imaging) are easy to perform due to limited complexity.

To improve the representation of the complex TME, monolayer cell cultures have been advanced into co-cultures enriched by coating with defined extracellular matrix (ECM) proteins or addition of distinct stem, stroma or (allogeneic) immune cell populations to allow the study of direct cell-cell interactions of different cell types or paracrine interactions in indirect cultures mostly using transwell inserts. The presence of immune cell populations (e.g. peripheral blood mononuclear cells (PBMC) or purified effector cells) is a prerequisite to study the preclinical effect of immunotherapies that aim to activate present immune cell populations. Alternatively, the cellular therapy itself (e.g. CAR T cells) constitutes the immune cell component within the co-culture model. Immunotherapeutic strategies including ICI (12), CAR T cells (1), CD3-targeted bispecific antibodies (24, 25) or oncolytic viruses (14) have been tested within 2D co-cultures. Of note, the cellular composition, activation and fitness status of circulating and tumor infiltrating immune cells often differs between healthy donors and cancer patients as they often display signs of reduced effector function and increased levels of exhaustion (26–30). Therefore, the integration of immune cells isolated directly from tumor tissue or PBMC of cancer patients into 2D cell cultures (as well as 3D co-culture models) is of great interest to approximate the functional capacity of the patient´s immune system. However, the use of allogeneic co-cultures to test respective immunotherapeutics is limited to a short experimental period up to a few days to avoid MHC-mediated alloreactions. Another critical point of this model system is that intratumor heterogeneity is not well reflected, as established tumor cell lines undergo clonal selection and genetic drift (31–33). Moreover, the complex tumor architecture with respect to spatial and cellular composition, ECM, gradients of oxygen, nutrients and other soluble factors including cytokines is obviously lacking (34, 35). Subsequently, these models have shown to have limited predictive value (36–38) as they do not optimally represent the complex tumor biology.

To improve the representation of patient’s tumors, primary tumor cells may be used instead. For example, Kodack et al. established mono cell cultures with primary cells isolated from tumor tissues of different tumor entities and advanced them into co-cultures with fibroblasts for drug testing of tyrosine kinase inhibitors (39). However, their success rate was limited to 26% with differing rates between tumor entities (39). Kornauth et al. demonstrated the potential of leukemia and lymphoma cell suspensions as a predictive tool for individualized treatment in aggressive hematologic malignancies (40). Within a clinical trial, single cell suspensions of tumor material (biopsies, blood or bone marrow aspirates) were generated and directly subjected to treatment with 139 drugs circumventing the time-consuming and failure-prone establishment of cancer cell lines. In this approach, the drug response of tumor cells within the cell bulk was determined by immunofluorescent microscopy and quantification of the surviving proportion of tumor cells in comparison to controls (40). Of note, 56 heavily pretreated patients were treated based on the results of this testing resulting in a clinical benefit in 54% (30 patients) including a relevant number of exceptional responses. Although these results are promising in terms of a co-clinical model, evaluation of immunotherapeutic strategies was not included in this trial and requires further advancement of this model by adding effector cell types or cellular therapies.

### 3D spheroids

A further improvement of the above mentioned 2D cultures are spheroids which are three dimensional aggregates of one or multiple cell types. Spheroids can be comprised of tumor cells (primary cells or cell lines) only or of mixtures of tumor and stroma/immune cells (41). Furthermore, ECM can be supplemented. The 3D structure results in formation of a hypoxic zone in the spheroid core as it is commonly observed in tumors where the tumor center is often hypoxic (42). Different culture techniques are used to generate spheroids, e.g. using low-adherent surface plates or the hanging drop method, but all of them are based on preventing attachment of tumor cells to the culture plate and promoting 3D cell-cell aggregation (43, 44). The fast and easy way to generate spheroids from established tumor cell lines along with established readout assays (45) allows high throughput drug screens which can be particularly beneficial for testing novel therapeutic approaches. Recently, 3D spheroids have been used to evaluate different immunotherapeutics, e.g. CAR NK cells against triple negative breast cancer (46), ICI targeting of PD-L1 in pancreatic ductal adenocarcinoma (PDAC) (41) or a strategy to activate tumor associated macrophages via CSF1R inhibition and CD40 activation in Her2-positive breast cancer (47).

However, besides most of the limitations mentioned for 2D cultures, the uncontrollable arrangement of the cells in the spheroids and the reduced complexity of the spheroids with regard to an incomplete cellular and acellular composition (42) limit the usage of 3D spheroids as co-clinical model particularly for testing immunotherapeutic strategies.

### Patient-derived organoids

Organoid technology has rapidly developed as a transformative 3D model since Clevers et al. established an intestinal 3D culture system from intestinal stem cells in 2009 (48). Organoid technology is now vastly used for modeling of physiological tissue but also of different cancers in patient-derived organoids (PDO). To generate organoids, small tissue fragments from surgical specimen or biopsies are dissociated into single cells and subsequently cultured, most often embedded in 3D matrices providing ECM support and in complex culture media enriched with multiple growth factors (49, 50). Today, PDO are available for multiple tumor entities, including prostate cancer (51), colorectal cancer (48, 52), or PDAC (53). Compared to 2D and spheroid cell cultures, PDO offer an improved insight into tumor biology as the heterogeneity of driver mutations and phenotypes of the primary tumor are better retained (53) and thus, tumor cell complexity, differentiation, and functionality are better represented (54, 55). Furthermore, PDO allow genetic engineering and genomic analyses that cannot be accurately modeled in animals (22, 56). However, major limitations of PDO remain the lack of vascularization and the complex TME (57) which sometimes constitutes the major compartment of a tumor, e.g. in cancers like PDAC (58, 59). Furthermore, time of establishment (currently weeks to months) is still time-consuming and the success rates are highly variable (16% to > 90%) differing between patients and tumor entities (60–64). Despite these limitations, PDO have been constantly advanced and increasingly used for preclinical testing of immunotherapies including ICI (7–9), bispecific antibodies (65), CAR T cells (66) or TIL generation (67).

For co-clinical evaluation, co-culture models of PDO with autologous immune cells and additional components of the TME appear to be ideal (68–70). To this end, Forsythe et al. established co-culture PDO models of appendiceal cancer with autologous immune cell populations to evaluate the efficacy of ICI nivolumab and pembrolizumab and identified 10-20% of PDO to be susceptible to ICI therapy (71). PDO may also offer a cost-effective opportunity to select for and expand TIL or generate patient specific cellular therapies. Dijkstra et al. successfully enriched autologous tumor reactive T cells from peripheral blood of colorectal and lung cancer patients (67). Similarly, Parikh et al. used organoids derived from metastases of multiple solid cancers to identify and generate TIL directed against individual tumor neoantigens with highly effective anti-tumor activity (72). These TIL co-cultured PDO could be established within two months for 75% of resected samples (72). To test CAR T cell treatment strategies in solid cancers, Schnalzger et al. used colorectal cancer PDO for evaluation of tumor cell killing and established a protocol to test the tumor cell specificity in competition assays using spiked-in organoids derived from healthy intestinal tissue (73). Beyond preclinical testing of immunotherapeutics, PDO can be employed to produce tumor cell specific T cells from induced pluripotent stem cells. This strategy may enable the production of allogeneic “off-the-shelf” CAR T cells circumventing the laborious and expensive generation of autologous CAR T cells (74). Moreover, large drug screens were successfully conducted implementing automated organoid seeding using automated microscopy or destructive viability assays as read-outs for drug efficacy paving the way for applications within the highly regulated clinical setting (75, 76). Recent studies indicate that PDO can be also used as co-clinical models for the prediction of treatment responses (60, 77, 78) and clinical trials are underway using functional testing in PDO to guide treatment decisions (79). Hence, several smaller collectives have been already established indicating a moderate to good correlation of drug responses in organoid-based patient avatar models with clinical responses (60, 77, 80). Guillen et al. combined mouse PDX and matched PDO of treatment resistant metastatic breast cancer to improve accuracy of modeling and combination of *in vitro* and *in vivo* drug testing (81).

However, to incorporate PDO-based treatment prediction into clinical workflows, PDO need to be improved in terms of reducing establishment time and optimizing generation success rates (61–64), highly varying among cancer patients and entities (60) not ensuring PDO generation from every patient. Finally, to accelerate meaningful implementation of PDO-based patient avatars into clinical application, prospective and systematic evaluation of their accurate representation of biological properties of the disease of origin and their predictive properties need to be considered in translational programs accompanying prospective clinical trials. Additionally, the implementation of the TME requires further developments, PDO generation needs to be methodologically standardized following standard operating procedures and predefined cut-offs for treatment response need to be defined to guide clinical decisions (82). Here, synthetic ECM substitutes have been already used to significantly reduce batch variability of ECM components ensuring a higher degree of standardization (83, 84). Hopes are high to use living PDO biobanks (52, 81, 85) for testing immunotherapies to build the translational bridge between basic research and patient care.

### Humanized patient-derived xenografts

To evaluate novel immunotherapies and identify biomarkers, humanized patient-derived xenografts (hPDX) are an important platform (86–92). Meanwhile, more than 45 PDX models are available including NSG, NOD-scid, NRG, BRGS, SRG and next-generation humanized mice (86, 89). Besides therapeutic responses, possible side effects as well as tumor progression and metastasis can be studied in these whole organism models. In hPDX, almost all histological, genetic, molecular, and immunological characteristics are at least represented at low passages (93, 94), fulfilling several key requirements of patient avatars (95–98). Particularly, testing of immunotherapies demands the patient’s immune system which can be activated towards the patient’s tumor. For this purpose, hPDX models require humanization of mice and full engraftment with the patient’s immune system. However, it has not been possible to reconstitute mice with the complete operational immune system of cancer patients, yet (86, 90, 91, 99). For testing immunotherapies based on T cells, the engraftment with patient’s PBMCs is of great interest. However, this is only feasible for short-term experiments due to the rapid onset of graft-versus-host disease (GvHD). This issue has been diminished by eliminating MHC-I and -II expression (100) or using mice lacking murine CD47 (101). Of note, PBMC-engrafted mice can undergo a switch in immune cellular composition within 7 days. As a result, T cells might dominate and concomitantly myeloid as well as B and NK cells are underrepresented (89) thereby not fully representing the immune system of patients. Alternatively, engraftment can be achieved by CD34+ human hematopoietic stem cells to study immunotherapies in hPDX (91, 102, 103).

An important limiting factor of hPDX as patient avatar is the generation duration of months up to a year (91) depending on tumor entity, technology and mouse strain (89). In most cases, this time frame is not feasible to establish a patient avatar as co-clinical model as patient´s treatment must start within a short period of time (i.e. most often within a few weeks, in cases with high tumor burden even faster). Additionally, it is necessary to take into account potential effects of patient’s pre-treatment in terms of acquired resistance mechanisms (104) or cumulative toxicity, which remains difficult to model in hPDX (105). Finally, even if tumors are transplanted with their respective human stroma, the TME in hPDX will be remodeled, e.g. by conversion from human to murine ECM (94).

Weighting the above-mentioned improvements and remaining limitations, good correlations between tumor responses in hPDX models and clinical responses of the corresponding patients were observed suggesting that this model is principally suitable as co-clinical model for therapy prediction (106–108). Moreover, hPDX have been used as major models to study CAR T cell therapies (2–4), ICI (5, 6, 11) and TIL (5).

### Organ-on-a-chip

As a strategy to avoid animal experiments, organ-on-a-chip (OOC) models have been designed to mimic physiological functions of different organs or tissues (109, 110). OOC can be based on established cell lines or organoids co-cultured with immune cells, fibroblasts or endothelial cells (111, 112). Additionally, epithelial and endothelial linings as well as ECM proteins may be included. In contrast to a conventional direct co-culture, in OOC cells are assembled on a chip containing a chamber and channels allowing for medium influx and efflux. Adding microfluidics via constant pumping of media allows to maintain gradients (e.g. of growth factors) and micromechanics (e.g. shear stress) while ensuring culture conditions for multiple cell types simultaneously. Geyer et al. modeled the physical barrier formed by pancreatic stellate cells (PSC) that prevent PBMCs, especially T cells, to migrate towards PDAC cells in a PDAC OOC. This barrier was overcome by treatment with Halofuginon inducing PSC death thereby increasing PBMC migration (113). These findings again illustrate the importance to consider the TME in the model system to properly test immunotherapeutic strategies. An additional layer of functional complexity can be added by including cell types that mediate drug metabolism, i.e. hepatocytes allowing the study of prodrugs (114). Cui et al. used a patient specific OOC to analyze the efficacy of anti-PD1 immunotherapy in different glioblastoma subtypes (10), Nyen et al. examined the response to trastuzumab and the impact of the tumor stroma in a breast cancer OOC (115) and Paterson et al. evaluated a CAR T construct in another breast cancer OOC (116). These studies clearly indicate the potential of OOC as patient avatar for individual therapy response prediction. Although OOC is a promising model for this purpose as it allows the combination of different cell types in one system and microscopic analysis is enabled by transparent polymers (109, 110), the most critical point is again the time needed for model establishment. Tumor cell isolation, organoid formation, OOC generation and treatment are all time-consuming steps, limiting its potential application as a patient avatar particularly for fast progressing and advanced cancers.

### Organotypic slice culture

Finally, patient derived organotypic slice cultures (OTSC) have emerged as a sophisticated patient avatar with a great potential to reduce the number of animal experiments (117, 118). OTSC are derived from tumor tissues obtained during surgical resection or via core needle biopsy (118–120). Afterward, tissues are cut mostly using a vibratom into tissue slices (117, 119, 120) The slice thickness varies from 150-500 µm and depending on tissue origin and cultivation method, OTSC remain intact for distinct time periods. Thus, it has been shown that OTSC remain viable for 5-9 days for PDAC (119–122), up to 10 days for non-small cell lung cancer (123), up to 6 days for breast cancer (124) and up to 16 days in glioblastoma (125). However, changes in the T(I)ME over time were not always characterized in detail (117, 119, 120, 122). Cultivation often takes place on inserts at the air-liquid-interface to ensure sufficient oxygenation and to prevent cell death due to hypoxia (120, 126). Here, the composition of the medium is a critical factor, as certain media can support growth of certain cell types and thus influence the original tissue composition by driving selection of certain cell clones and phenotypes (119, 127).

In contrast to organoids and other cell culture models, which often represent only a reconstruction of the original tumor cell compartment, OTSC preserve the tumor and stroma heterogeneity thereby representing the tumor in its native environment, comprising epithelial/tumor cells, entire ECM as well as stroma and immune cells (117, 121, 128). In this way, all cells retain their function (hormone secretion, vascular contractility, cytokine secretion) along with their proteome and secretome (e.g. for immunological functions), and neurons also remain viable due to the presence of nerve growth factor (117, 129). This high similarity to the original tumor tissue creates unique conditions for analyzing the interplay of tumor cells with their TME thereby providing improved insights into tumor biology. Embedding of glioblastoma spheroids in brain tissue slices, Decotret et al. showed that the brain TME has a decisive influence on glioblastoma cell invasion (130). Besides, OTSC also appear to be well suited for the development and testing of novel therapeutic approaches (117, 118, 131). Thus, a combination treatment targeting carcinoma associated fibroblasts (CAF) by CXCR4 blockade and immune cells by ICI, increased T cell migration and activation towards tumor cells was observed resulting in tumor cell apoptosis (118). In line with these results, ECM reduction in OTSC improved T cell invasion towards tumor cells and increases the efficacy of blockade of the immune checkpoint molecule PD-1 (13). As OTSC are the only model system preserving the entire patient’s tumor contexture over a distinct time period, it can be considered the best patient avatar to date. Importantly first studies indicate that OTSC are suitable for testing immunotherapies (123), although data on the correlation between treatment responses in patients and corresponding OTSC is still scarce. Therefore, the predictive power of OTSC has to be proven yet.

Besides these important advantages of OTSC, some critical points still deserve optimization. As mentioned above, the medium composition impacts survival and proliferation of cells thereby selecting certain cell populations (127). Furthermore, despite cultivation at the air-liquid-interface, longer cultivation might lead to hypoxia resulting in culture-induced cell death in certain areas of the section (120, 126). The limited culture duration in turn also impedes long-term studies including studies analyzing long-term effects of applied treatments. Furthermore, long-term storage of viable OTSC for future analyses is not possible yet, and the limited number of OTSC which can be obtained from one patient limits high throughput drug screening (120). Finally, to properly assess treatment responses, appropriate and reliable readout parameters have to be identified and quantified. Here, (live cell) imaging might be difficult due to the thickness of the OTSC (117).

However, since the response to (immuno)therapies often varies among cancer patients, OTSC have a high potential to play a role in the development of patient tailored therapy. The rapid availability of OTSC after surgery or core needle biopsy allows for rapid drug testing and, at the same time, characterization of the entire tumor including its T(I)ME even in patients with advanced tumor diseases. This offers the great opportunity to allow a prompt therapy prediction for the patient.

## Discussion and future perspectives

Significant progress has been made advancing existing *in vitro*, *ex vivo* and *in vivo* tumor models into patient avatars containing the patient´s T(I)ME thereby trying to mimic the patient´s tumor characteristics in the best possible manner. These efforts have led to invaluable insights into tumor (immune) biology and the efficacy of immunotherapeutic strategies. However, as outlined above and summarized in **Table 1**, every model system bears its advantages and disadvantages which need to be carefully weighed in order to make the right choice of the patient avatar for research or co-clinical therapy testing and prediction. Accordingly, further efforts are needed to focus on the following two aspects: First, addressing remaining limitations in the representation of the T(I)ME in existing models and second, advancing existing models towards co-clinical patient avatars that support clinical decision making based on functional assays. Results from these assays may then complement existing strategies to personalize tumor therapies based on genomics, transcriptomics and immunohistochemical tumor analysis.

**Table 1:** Overview of key features of currently used patient avatars and their suitability as co-clinical models for testing of immunotherapies.

	*in vitro* – 2D	*in vitro* – 3D			*ex vivo*	*in vivo*
	Cell models	Spheroids	PDO	OOC	OTSC	hPDX
**Tumor cell heterogeneity**	limited for established cell lines	limited for established cell lines	improved	improved	high	high
**Microenvironment**	ECM has to be exogenously added, indirect & direct co-cultures with allogeneic immune or stroma cell populations possible	ECM has to be exogenously added, direct co-cultures with allogeneic immune or stroma cell populations possible	ECM has to be exogenously added, indirect & direct co-cultures with allogeneic (autologeous) immune or stroma cell populations possible	ECM has to be exogenously added, Indirect & direct co-cultures with allogeneic (autologeous) immune or stroma cell populations possible	completely preserved for distinct time (depending on tumor entity)	completely preserved for distinct time, conversion into murine stroma
**Nutrient/oxygen gradient & vascularization**	missing	hypoxic zone in spheroid core, lack of vascularization	missing	possible	nutrient & oxygen gradient observed, lack of vascularization	present
**Model establishment**	fast	fast	time consuming	time consuming	fast	time consuming
**Reproducibility**	high	variable	patient-dependent	variable	patient-dependent	patient-dependent
**High throughput screening**	possible	possible	possible	limited	limited	limited
Testing of immunotherapies*	ICI (12) Bispecific antibodies (24, 25) CAR T cells (1) Oncolytic viruses (14)	CAR-NK cells (46) ICI (41) Macrophage activation (47)	Therapy prediction (60, 77, 78) ICI (7–9, 71) Bispecific antibodies (65) CAR T cells (66, 73, 74) TIL (67, 72)	ICI (10) CAR T cells (116)	Drug testing & development (117, 118, 131) ICI (13, 118)	therapy prediction (81, 96, 106–108) CAR T cells (2–4) ICI (6, 11) TIL (5)

*Only exemplary studies mentioned in the text are listed.

Finally, to advance patient avatars towards clinical application, a critical and important point is the standardization, e.g. by using harmonized protocols for generation and maintenance, reducing batch variability in reagents, increasing throughput while reducing costs for generation and characterization and defining experimental endpoints that are clinically meaningful (82, 132). Organoid or OTSC-based models may then even serve to develop patient specific therapies such as TIL and CAR T cells.

The current dynamics of the field are reflected by a multitude of ongoing clinical trials set up to evaluate the power of organoid or PDX based-models to predict clinical outcomes in cancer patients (133). Results from these mostly observational clinical trials will provide novel insights into feasible strategies to advance and implement personalized functional assays based on patient avatars for evaluation of (immuno) therapeutics.

## Data availability statement

The original contributions presented in the study are included in the article/**Supplementary Material**. Further inquiries can be directed to the corresponding author.

## Author contributions

Conceptualization, CK, AB, AW, SS. Supervision, SS. Visualization, CK, AB. Writing – original draft, AB, CK, AW, SS. Writing – review and editing, AB, CK, AW, SS. All authors contributed to the article and approved the submitted version.
