# Supplementary material for: The challenge of making the right choice: patient avatars in the era of cancer immunotherapies
Source: Front Immunol. 2023 Aug 10;14:1237565. doi: 10.3389/fimmu.2023.1237565 (PMC10449253; doi:10.3389/fimmu.2023.1237565)
Supplement: Supplementary file 1 [file Presentation_1.pptx]

## Slide 1
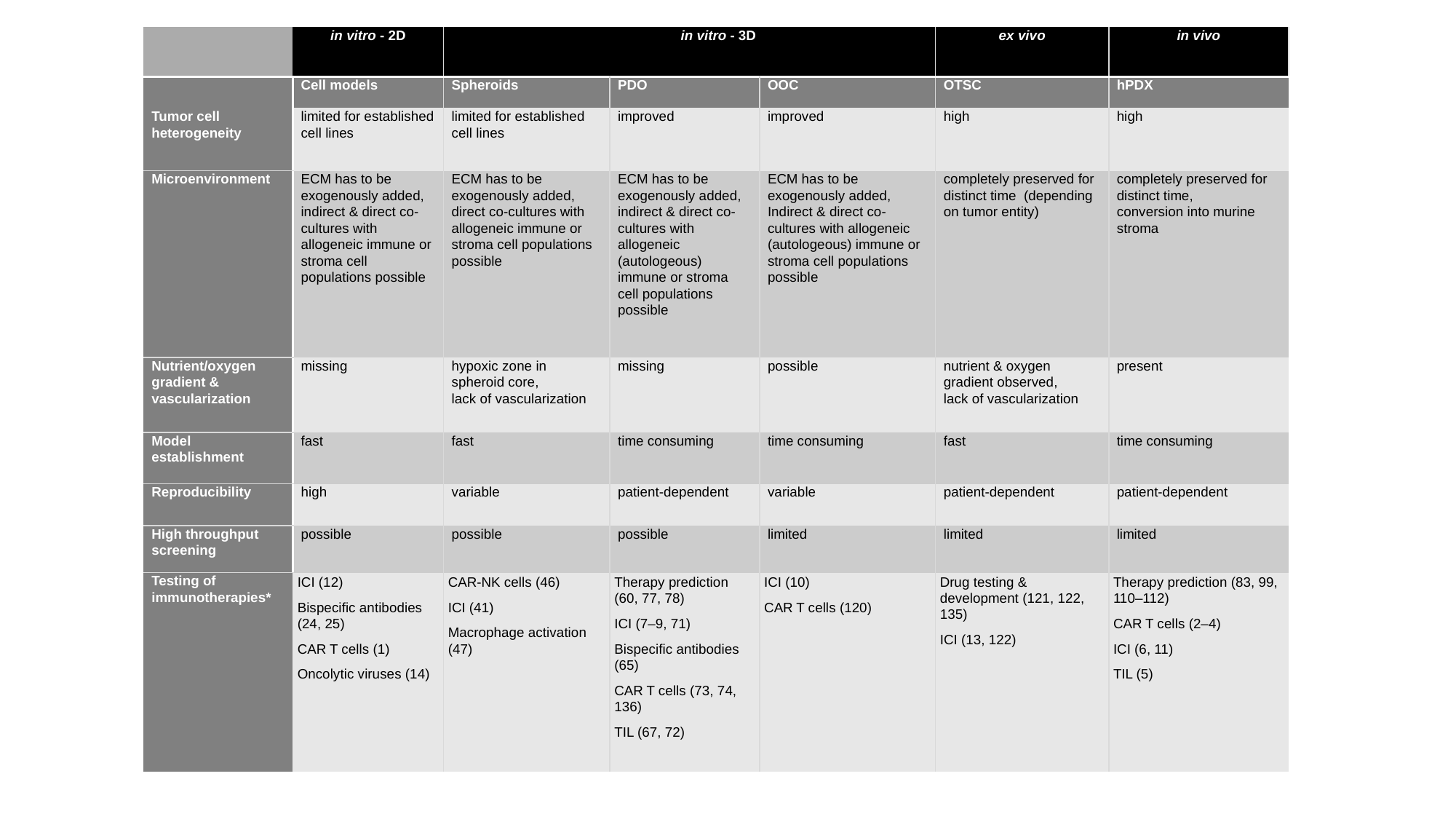

| | in vitro - 2D | in vitro - 3D | | | ex vivo | in vivo |
| --- | --- | --- | --- | --- | --- | --- |
| | Cell models | Spheroids | PDO | OOC | OTSC | hPDX |
| Tumor cell heterogeneity | limited for established cell lines | limited for established cell lines | improved | improved | high | high |
| Microenvironment | ECM has to be exogenously added, indirect & direct co-cultures with allogeneic immune or stroma cell populations possible | ECM has to be exogenously added, direct co-cultures with allogeneic immune or stroma cell populations possible | ECM has to be exogenously added, indirect & direct co-cultures with allogeneic (autologeous) immune or stroma cell populations possible | ECM has to be exogenously added, Indirect & direct co-cultures with allogeneic (autologeous) immune or stroma cell populations possible | completely preserved for distinct time (depending on tumor entity) | completely preserved for distinct time, conversion into murine stroma |
| Nutrient/oxygen gradient & vascularization | missing | hypoxic zone in spheroid core, lack of vascularization | missing | possible | nutrient & oxygen gradient observed, lack of vascularization | present |
| Model establishment | fast | fast | time consuming | time consuming | fast | time consuming |
| Reproducibility | high | variable | patient-dependent | variable | patient-dependent | patient-dependent |
| High throughput screening | possible | possible | possible | limited | limited | limited |
| Testing of immunotherapies\* | ICI (12) Bispecific antibodies (24, 25) CAR T cells (1) Oncolytic viruses (14) | CAR-NK cells (46) ICI (41) Macrophage activation (47) | Therapy prediction (60, 77, 78) ICI (7–9, 71) Bispecific antibodies (65) CAR T cells (73, 74, 136) TIL (67, 72) | ICI (10) CAR T cells (120) | Drug testing & development (121, 122, 135) ICI (13, 122) | Therapy prediction (83, 99, 110–112) CAR T cells (2–4) ICI (6, 11) TIL (5) |
